# Triptolide improves neurobehavioral functions, inflammation, and oxidative stress in rats under deep hypothermic circulatory arrest

**DOI:** 10.18632/aging.202460

**Published:** 2021-01-19

**Authors:** Qiang Chen, Yu-Qing Lei, Jian-Feng Liu, Zeng-Chun Wang, Hua Cao

**Affiliations:** 1 Department of Cardiac Surgery, Fujian Maternity and Child Health Hospital, Affiliated Hospital of Fujian Medical University, Fuzhou 350001, P. R. China

**Keywords:** triptolide, deep hypothermia circulatory arrest (DHCA), neuroinflammation, oxidative stress, neurotrophins

## Abstract

This study investigated the neuroprotective effects of triptolide (TPL) in a rat model of cardiopulmonary bypass with deep hypothermia circulatory arrest (DHCA). Rats were randomly divided into six groups: control, sham, DHCA, and DHCA + TPL (100, 200, 300 μg/kg). Neurobehavioral functions were measured using the elevated plus-maze, Y-maze, and Morris water maze tests. Levels of inflammatory cytokines, oxidative stress indices, and brain neurotrophins were measured by ELISA. Microglial activation and cell death was measured by immunofluorescence staining and TUNEL assay, respectively. Finally, activation of the Nrf2 pathway and NF-κB were detected by western blot. The elevated plus-maze, Y-maze, and Morris water maze tests all showed that TPL mitigated anxiety-like behavior, working memory, spatial learning, and memory in DHCA rats. TPL inhibited inflammatory responses and oxidative stress, as well as increased brain neurotrophin levels in DHCA rats. Moreover, TPL attenuated microglia activation and cell death in DHCA rats. Finally, TPL activated the Nrf2 pathway and inhibited NF-κB activity in DHCA rats. These results demonstrated that TPL improved neurobehavioral functions, neuroinflammation, and oxidative stress in DHCA rats, which may be associated with the Nrf2 and NF-κB pathways.

## INTRODUCTION

Cardiopulmonary bypass (CPB) with deep hypothermia circulatory arrest (DHCA) is a technique that supports vital organs and is often used during the repair of complex neonatal congenital heart, adult congenital heart, and aortic arch diseases. DHCA provides a relatively bloodless field to facilitate the repair of complex congenital or acquired pathologies during cardiac surgery. However, it contributes to profound perturbations in inflammatory and oxidative stress effects, which are collectively implicated in the pathogenesis of perioperative cerebral injury [1–3]. Therefore, it is necessary to understand the pathophysiology of DHCA to reduce its neurological complications.

The NF-κB pathway is a critical regulator of neuroinflammation-associated disease pathogenesis [4]. Our previous study demonstrated that the synthesis of pro-inflammatory cytokines such as IL-6, IL-1β, and TNF-α after DHCA was mediated through NF-κB-dependent signaling [5]. Accumulating evidence has shown that oxidative stress plays a pivotal role in cerebral ischemia and reperfusion (I/R) injury [6, 7]. Nuclear factor E2–related factor 2 (Nrf2) is one of the key modulators of defense against oxidative stress. Nrf2 binds to antioxidant response element (ARE) sequences, upregulating the antioxidant enzyme haemoxygenase-1 (HO-1) [8, 9]. This suggests that the Nrf2 pathway may play an important role in protecting the brain from I/R injury. Therefore, it may be feasible to alleviate DHCA-induced brain injury by regulating the NF-κB and Nrf2 pathways to inhibit inflammation and oxidative stress, respectively.

Over the past decade, the neuroprotective effects of phytochemicals have been extensively studied. Triptolide (TPL) is an active compound derived from *Tripterygium wilfordii* [10]. Studies have shown that TPL is a candidate neuroprotective agent that can improve neurodegenerative diseases by reducing the production of inflammatory cytokines and alleviating oxidative stress. TPL has been shown to reduce oxidative stress and improve cognitive impairment in a rat model of vascular dementia [11, 12]. It was also reported that TPL exerts anti-inflammatory and antioxidative effects in a transgenic mouse model of I/R injury through anti-inflammatory and antioxidant functions [13–15]. However, the beneficial effects of TPL on DHCA-induced cerebral injury have not been studied. This study investigated the neuroprotective effects of TPL on a rat DHCA model. We hypothesized that TPL could inhibit inflammation and oxidative stress by regulating the NF-κB and Nrf2 pathways, thereby reducing cerebral damage caused by DHCA. Our study provides novel insights into the *in vivo* mitigation of neurological damage following DHCA by TPL.

## RESULTS

### TPL improved the neurobehavioral functions of DHCA model rats

**Figure 1:**
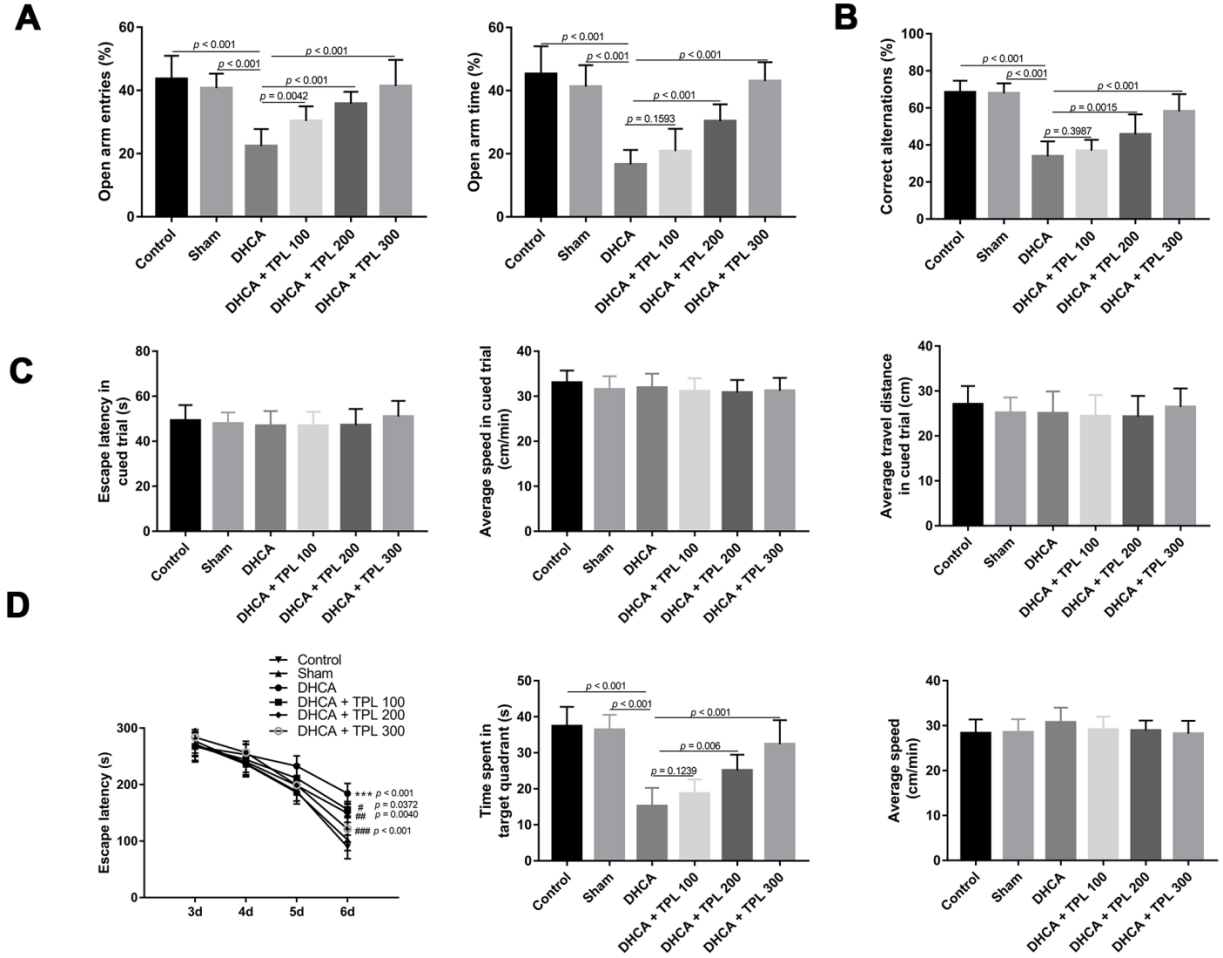
**TPL improved neurobehavioral functions of rats after DHCA.** On day 3 after DHCA, rats underwent elevated plus-maze test (**A**) and Y maze test (**B**) to evaluate anxiety-like behavior and working memory. TPL treatment increased the percentage of open arms entries and open arms time after DHCA. From day 3 through day 7 after DHCA, rats underwent Morris water maze test to evaluate spatial learning and memory. There was no difference in escape latency, speed and travel distance among groups in the cued phase (**C**). The results showed that TPL treatment significantly shortened the escape latency and increased the time spent in goal quadrant compared to DHCA group (**D**). Values were presented as $\bar{x}\pm s$ (n = 10). The MWM data was analyzed by repeated measures ANOVA, with time as the repeated measure and Fisher’s least significance difference post hoc test. Parametric values were analyzed by one-way ANOVA followed by Bonferroni's multiple comparison tests. Kruskal-Wallis test followed by Dunn’s multiple comparison was used to analyze nonparametric values. A difference with *P* < 0.05 was indicated statically significant. ***P < 0.001 compared to the intact control group and sham group; ##*P* < 0.01, ###*P* < 0.001 compared to the DHCA group.

In the EPM test, the percentage of open-arm entries and the open-arm time of rats subjected to DHCA were decreased compared with the intact control and sham groups (*P*<0.001). However, 100 μg/kg, 200 μg/kg, and 300 μg/kg TPL treatment increased the percentage of open-arm entries compared with the DHCA group (*P*<0.01–0.001). Furthermore, 200 μg/kg and 300 μg/kg TPL increased the percentage of open-arm time compared with the DHCA group (*P*<0.001) (Figure 1A).

Results of the Y-maze test showed that DHCA decreased the percentage of correct alternations compared with the intact control and sham groups (*P*<0.001). TPL treatments of 200 μg/kg and 300 μg/kg significantly increased the percentage of correct alternations compared with the DHCA group (*P*<0.001) (Figure 1B).

In the MWM test, there was no difference in escape latency, speed, or travel distance among the groups in the cued phase (Figure 1C). The navigation test showed that the escape latency of each group decreased with increasing number of training days. Compared with the DHCA group, the escape latencies of the different TPL treatments were significantly shortened (*P*<0.05–0.001). On day 7, the space exploration test indicated that the time spent in the target quadrant of rats subjected to DHCA was reduced compared with the intact control and sham groups (P<0.001). In contrast, rats treated with TPL at doses of 200 μg/kg and 300 μg/kg spent more time in the goal quadrant (*P*<0.01–0.001). The swimming speeds of each group were not significantly different (Figure 1D).

### TPL inhibited DHCA-induced inflammatory responses in model rats

**Figure 2:**
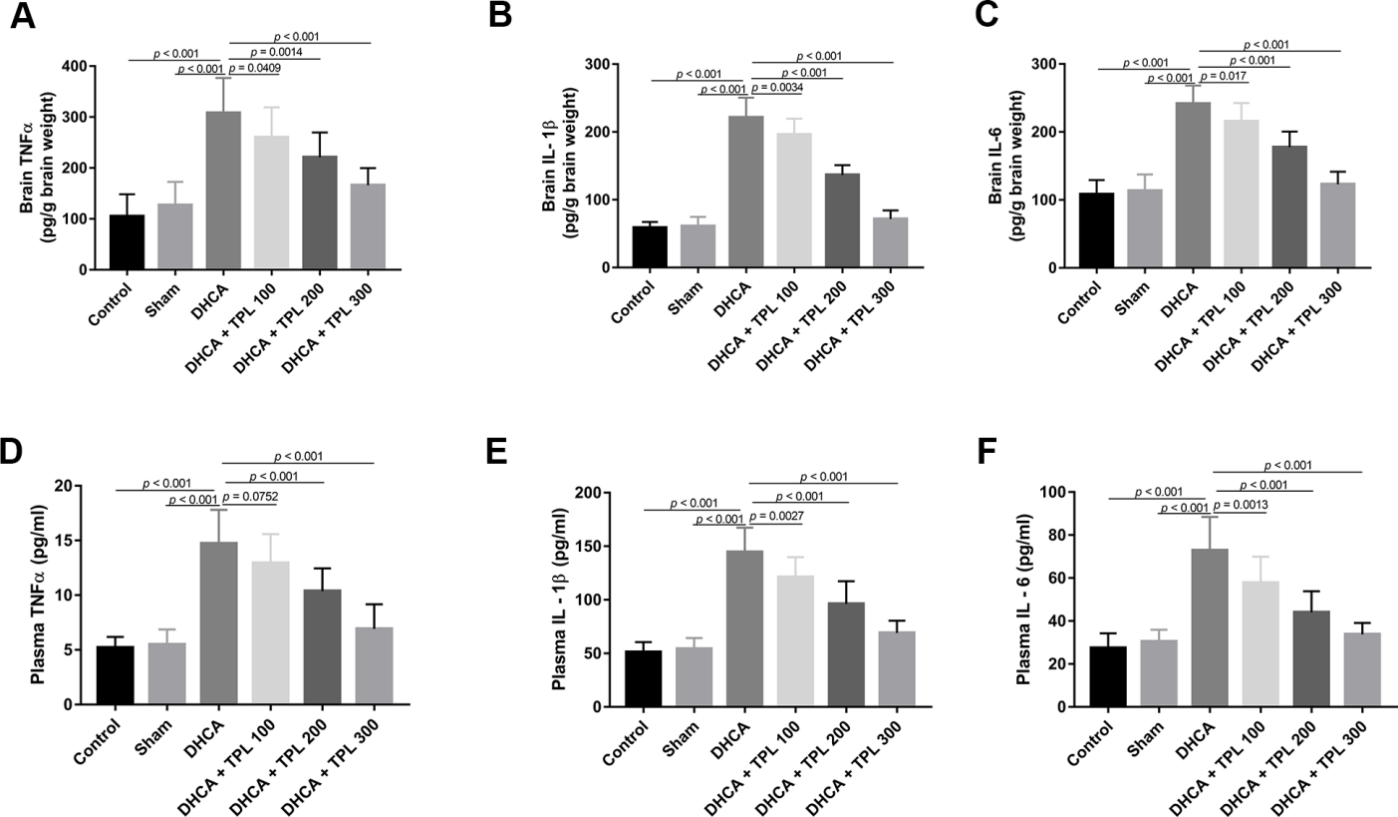
**TPL inhibited DHCA induced inflammatory response in rats.** On day 7 post operation, rats were euthanized and collected plasma and brain tissues to detect the inflammatory cytokines levels. The levels of brain TNFα (**A**), IL-1β (**B**), IL-6 (**C**) were decreased with TPL treatment after DHCA. In line with the results of brain, TPL treatment reduced the elevation of TNF-α (**D**), IL-1β (**E**) and IL-6 (**F**) levels in plasma after DHCA. Values were presented as $\bar{x}\pm s$ (n = 10). A difference with *P* < 0.05 was indicated statically significant. One-way ANOVA followed by Bonferroni's multiple comparison tests was performed to analyze differences between groups.

After DHCA, the inflammatory cytokines TNF-α, IL-1β, and IL-6 were increased in the brain (*P*<0.001), and different doses of TPL treatment decreased their levels compared with the DHCA group (*P*<0.05–0.001) (Figure 2A–2C). Consistent with results from the brain, TPL treatment also mitigated the increased levels of TNF-α, IL-1β, and IL-6 in plasma after DHCA (*P*<0.01–0.001) (Figure 2D–2F).

### TPL inhibited DHCA-induced oxidative stress in model rats

**Figure 3:**
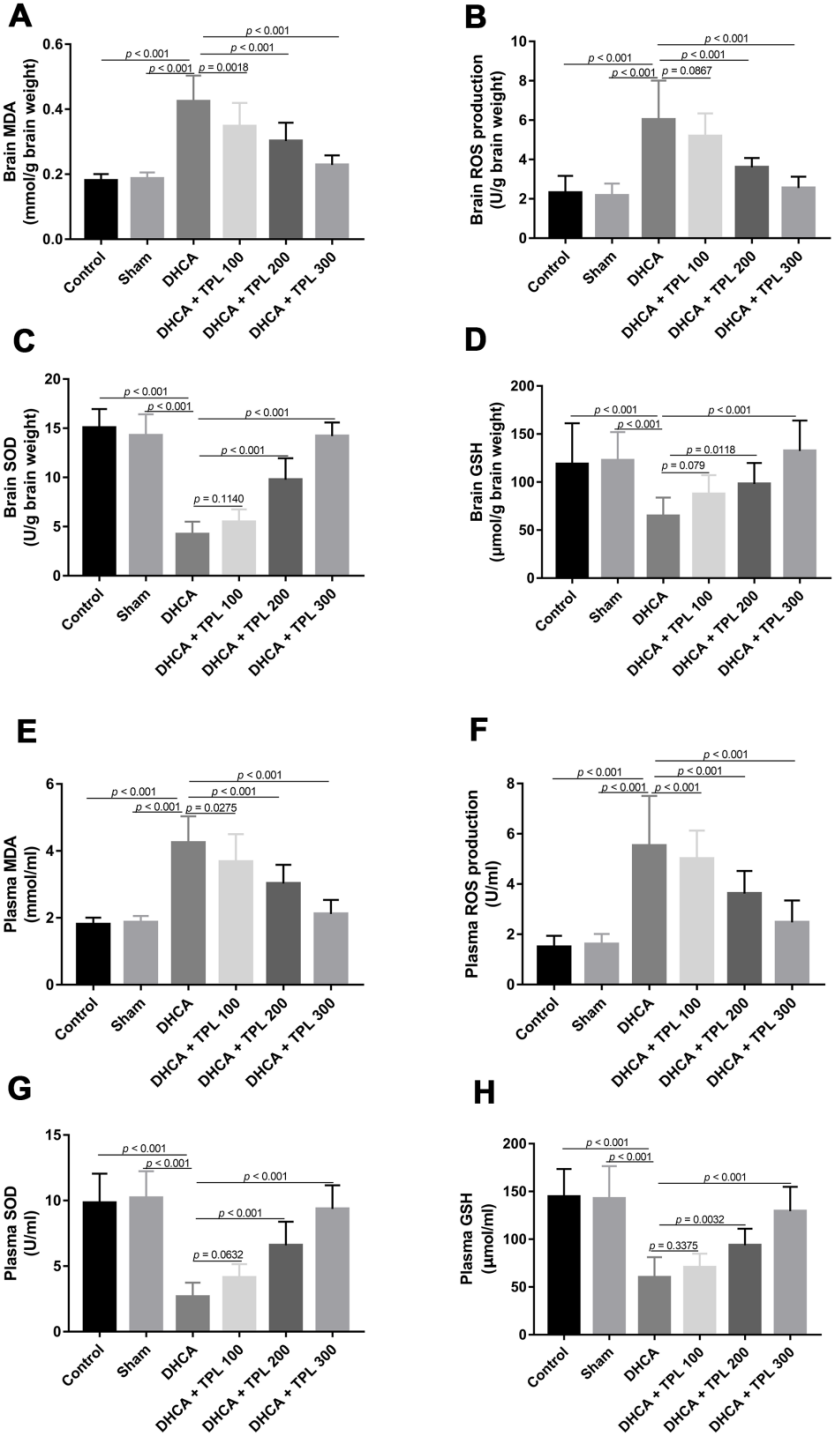
**TPL inhibited DHCA induced oxidative stress in rats.** On day 7 post operation, rats were euthanized and collected plasma and brain tissues to evaluate oxidative stress. The levels of brain MDA (**A**) and ROS production (**B**) were decreased with TPL treatment after DHCA. The activity of brain SOD (**C**) and GSH (**D**) was increased with TPL treatment after DHCA. Meanwhile, TPL treatment reduced plasma MDA (**E**) and ROS production (**F**) levels and increased the activities of SOD (**G**) and GSH (**H**) after DHCA. Values were presented as $\bar{x}\pm s$ (n = 10). Values were presented as $\bar{x}\pm s$ (n = 10). A difference with *P* < 0.05 was indicated statically significant. One-way ANOVA followed by Bonferroni's multiple comparison tests was performed to analyze differences between groups.

We next examined the effect of TPL on the oxidative stress induced by DHCA. Compared with the intact control and sham groups, the production of malondialdehyde (MDA) and reactive oxygen species (ROS) were increased and superoxide dismutase (SOD) and Glutathione (GSH) activity were reduced in brain tissues of rats subjected to DHCA (*P*<0.001). TPL inhibited the production of MDA and ROS, and elevated SOD and GSH activity (*P*<0.05–0.001) (Figure 3A–3D). Next, we measured the oxidative stress in circulation. Plasma levels of MDA and ROS were markedly elevated, and the activity of SOD and GSH were decreased in the DHCA group compared with the intact control and sham groups (*P*<0.001). TPL significantly reduced levels of ROS and MDA, and increased levels of SOD and GSH (*P*<0.05–0.001) (Figure 3E–3H).

### TPL increased brain neurotrophins in DHCA model rats

**Figure 4:**
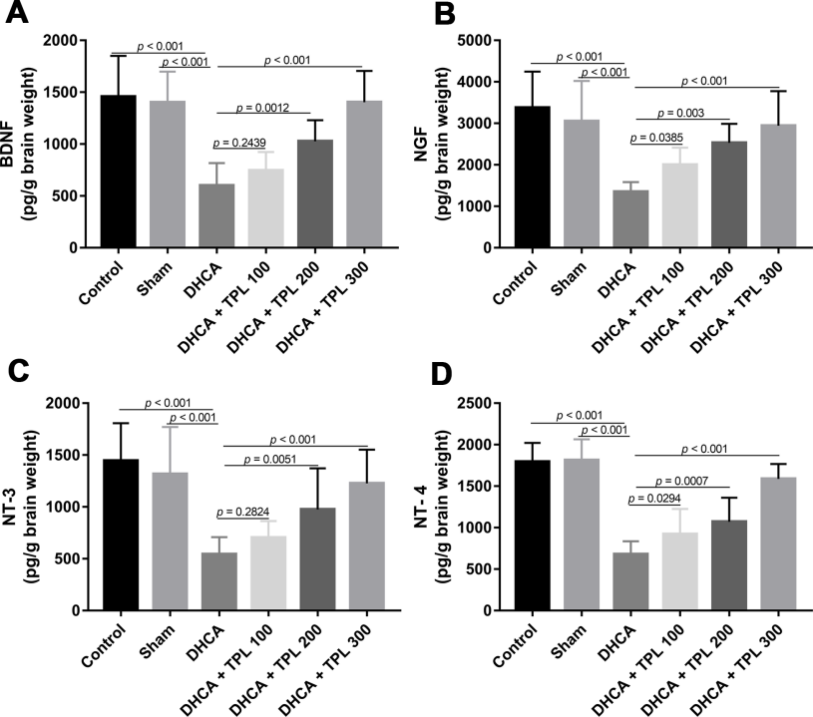
**TPL increased brain neurotrophins in DHCA rats.** On day 7 post operation, rats were euthanized and collected brain tissues to measure brain neurotrophins levels. The levels of brain BDNF (**A**), NGF (**B**), NT-3 (**C**) and NT-4 (**D**) were increased with TPL treatment after DHCA. Values were presented as $\bar{x}\pm s$ (n = 10). A difference with *P* < 0.05 was indicated statically significant. One-way ANOVA followed by Bonferroni's multiple comparison tests was performed to analyze differences between groups.

As shown in Figure 4, levels of the brain neurotrophins BNDF, NGF, NT-3, and NT-4 were decreased in rats subjected to DHCA (*P*<0.001). In contrast, TPL treatment restored the BNDF, NGF, NT-3 and NT-4 levels compared with the DHCA group in a dose dependent manner (*P*<0.05–0.001).

### TPL attenuated microglia activation and cell death in DHCA model rats

**Figure 5:**
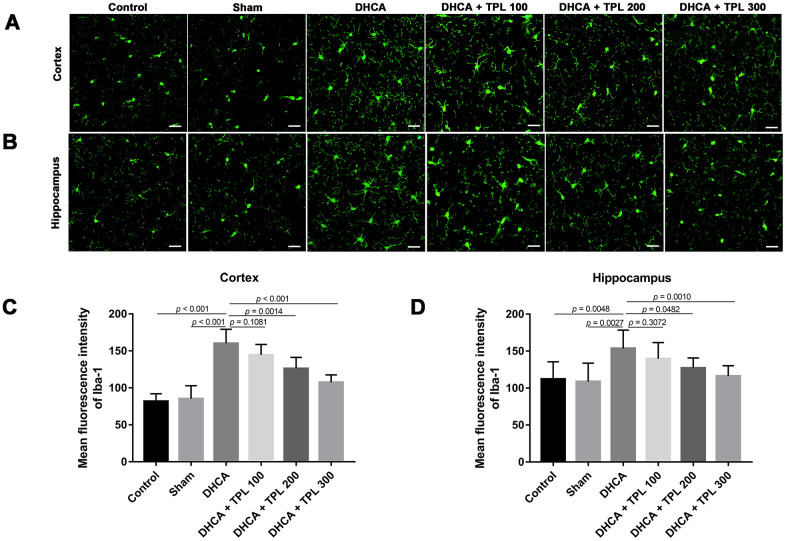
**TPL attenuated the activation of microglia in DHCA rats.** The effect of TPL on the activation of microglia in cortex (**A**) and hippocampus (**B**) after DHCA was detected by immunofluorescence assay. TPL effectively inhibited microglial activation both in cortex (**C**) and hippocampus (**D**) after DHCA. Values were presented as $\bar{x}\pm s$ (n = 10). A difference with *P* < 0.05 was indicated statically significant. One-way ANOVA followed by Bonferroni's multiple comparison tests was performed to analyze differences between groups.

**Figure 6:**
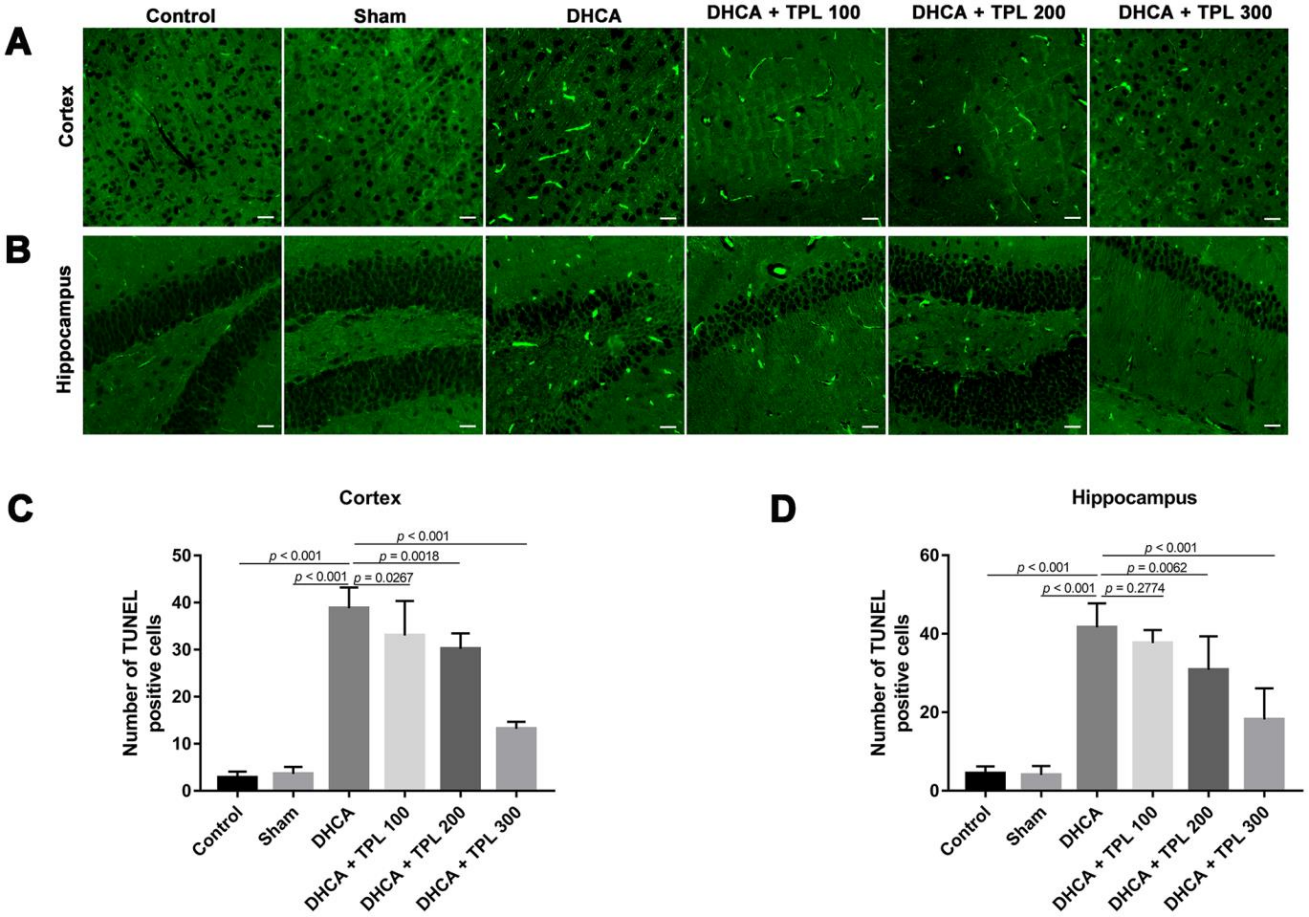
**TPL attenuated cell death in the hippocampus and cortex in DHCA rats.** The effect of TPL on the cell death in cortex (**A**) and hippocampus (**B**) after DHCA was detected by TUNEL assay. TPL significantly reduced cell death both in cortex (**C**) and hippocampus (**D**) after DHCA. Values were presented as $\bar{x}\pm s$ (n = 10). A difference with *P* < 0.05 was indicated statically significant. One-way ANOVA followed by Bonferroni's multiple comparison tests was performed to analyze differences between groups.

Microglia activation is a critical hallmark in neuroinflammation. After DHCA, a significant activation of microglia was found, while the 200 μg/kg and 300 μg/kg TPL treatments remarkably mitigated microglia activation after DHCA (*P*<0.05–0.001) (Figure 5). TPL treatment was also associated with a significant reduction in TUNEL-positive cells in the cortex and hippocampus after DHCA (*P*<0.05–0.001) (Figure 6).

### TPL activated the Nrf2/NQO-1/HO-1 pathway and inhibited NF-κB p65 activity in DHCA model rats

**Figure 7:**
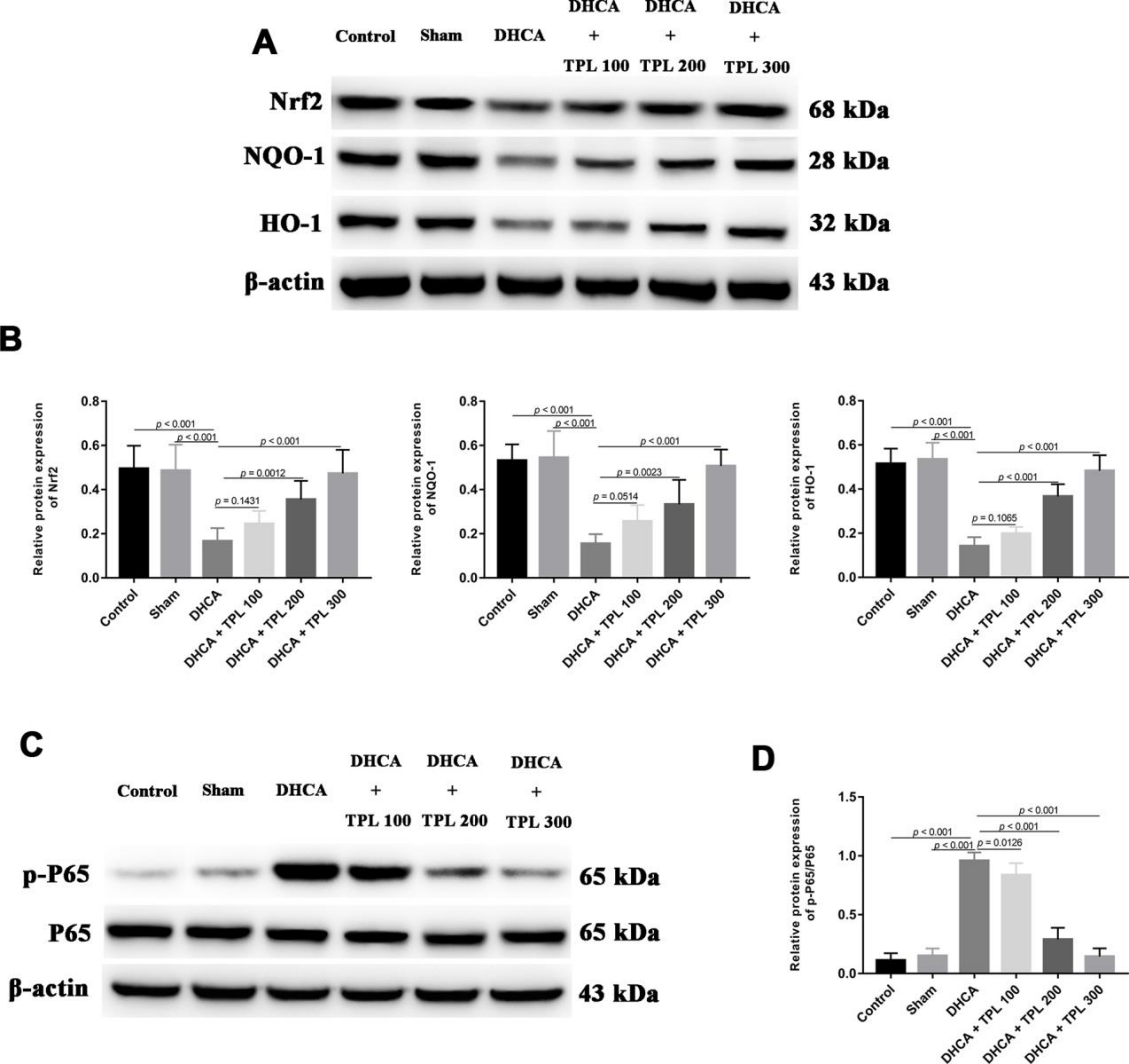
**TPL activated Nrf2/NQO-1/HO-1 pathway and inhibited the activation of NF-κB p65 in DHCA rats.** The expression of Nrf2 pathway and activation of NF-κB p65 were measured using western blot analysis. The western blots and bar graph summarized data showed that TPL treatment elevated Nrf2 pathway (**A**, **B**) and suppressed NF-κB p65 activation (**C**, **D**) after DHCA. Values were presented as $\bar{x}\pm s$ (n = 10). A difference with *P* < 0.05 was indicated statically significant. One-way ANOVA followed by Bonferroni's multiple comparison tests was performed to analyze differences between groups.

By measuring the levels of Nrf2/NQO-1/HO-1 pathway members and of NF-κBp65, the potential molecular mechanism of TPL against DHCA-induced inflammation and oxidative stress was detected. Compared with the intact control and sham groups, the Nrf2 pathway was significantly inhibited in DHCA rats (*P*<0.001). In contrast, 200 μg/kg and 300 μg/kg TPL treatments upregulated the Nrf2 pathway in DHCA rats (*P*<0.05 or *P*<0.001) (Figure 7A, 7B). Moreover, the p-P65/P65 ratio was remarkably increased in the DHCA group (*P*<0.001), but all TPL treatments inverted the p-P65/P65 ratio compared with the DHCA group (*P*<0.001) (Figure 7C, 7D).

## DISCUSSION

The results of this study demonstrated that 100–300 μg/kg TPL treatments could significantly improve neurobehavioral functions and inhibit inflammation and oxidative stress in rats after DHCA. The Nrf2 pathway and NF-κB p65 activity were associated with the anti-inflammatory and antioxidative effects of TPL in DHCA rats.

It has been reported that more than half of patients who receive DHCA treatment suffer from postoperative neurological dysfunction [3]. Rats subjected to DHCA showed impaired cognitive outcomes [16, 17]. In the MWM test, DHCA induced impairments in spatial learning and memory, that manifested by extended escape latency and reduced crossing to target platforms [18]. TPL is a biologically active compound derived from herbal medicines that has been regarded as a drug candidate to alleviate neurodegenerative diseases [19–23]. It has been reported that TPL attenuated neurological deficits in I/R rats and rats with ischemic stroke [13, 14]. In parallel with these studies, we showed that TPL treatment improved the spatial learning and working memory of rats after DHCA in the MWM and Y maze tests. It was also reported that anxiety and depression symptoms are associated with I/R injury [24, 25]. Our study also revealed that anxiety-like behaviors increased after DHCA, and that TPL decreased anxiety-like behaviors in from DHCA.

Patients receiving DHCA had increased circulating inflammatory cytokines [26]. In a rat DHCA model, blood TNF-α and IL-6 levels were elevated [27]. It is well known that neuroinflammation after cardiac surgery is affected by systemic inflammation, which is manifested by increased inflammatory cytokines in circulation and spinal fluid [28–31]. Our study showed that levels of the pro-inflammatory cytokines IL-6, IL-1β, and TNF-α were increased in both plasma and brain tissue after DHCA. Moreover, TPL treatment not only reduced the levels of pro-inflammatory cytokines in circulation, but also affected the brain concentrations of corresponding inflammatory cytokines in DHCA rats. Microglia are the native marrow cells in the brain that produce pro-inflammatory mediators such as TNF-α, IL-1β, IL-6, ROS, and NO after an I/R injury [32]. Our results of immunofluorescence staining of microglia found that TPL treatment remarkably mitigated microglia activation after DHCA, which further confirmed the anti-neuroinflammatory effects of TPL.

The pathophysiology of DHCA primarily consists of systemic inflammation induced by contact of the blood with an internal surface of the cardiopulmonary bypass tube and oxidative stress caused by I/R injury [33]. A previous study showed that DHCA rats had significantly increased levels of oxidative stress biomarkers, such as MDA and 8-Hydroxydeoxyguanosine (8-OH-dG), and decreased levels of the antioxidant SOD in circulation and cerebral tissue [34]. In line with this, we found that DHCA induced oxidative stress in the plasma and brain with increased production of oxidants MDA and ROS and decreased antioxidants SOD and GSH levels. Zhou et al [35] found that 200 μg/kg TPL inhibited the circulating oxidative stress by reducing MDA levels and enhancing SOD activity in rats with membranous nephropathy. In vascular dementia rats, TPL treatment significantly increased SOD activity and decreased MDA activity in the hippocampus. Additionally, TPL protected PC12 cells from the oxidative stress induced by Aβ_25–35_ by decreasing ROS and MDA levels. Similar to the above findings, our data demonstrate that TPL significantly reduced oxidant levels of ROS and MDA, and increased antioxidant levels of SOD and GSH compared with the DHCA group in the plasma and brain.

BDNF, NGF, NT3, and NT-4 are neurotrophin family members [36]. Elevated neurotrophin levels in the brain can exert a neuroprotective effect against ischemia damage [37–39]. Our study also demonstrated that levels of the neurotrophic factors BDNF, NGF, NT3, and NT-4 were restored by TPL treatment in DHCA rats. Therefore, it is possible that TPL improved neurological deficits in DHCA rats by increasing neurotrophin levels.

To investigate the molecular mechanisms underlying the neuroprotective effects of TPL in DHCA rats, we measured the Nrf2 pathway and NF-κB p65 activity. Nrf2 signaling has been shown to be involved in oxidative stress in rats with brain I/R injury [40]. Nrf2 exerts an antioxidant effect by regulating the downstream enzymes NQO-1 and HO-1 [41]. Previous studies have found that chlorogenic acid had antioxidative effects on rats with brain I/R injury by mediating the Nrf2/NQO-1/HO-1 pathway [39]. Similarly, this study showed that the Nrf2/NQO-1/HO-1 pathway was inhibited in DHCA rats, while TPL reactivated Nrf2 signaling, which may further elevate SOD and GSH activity and decrease MDA and ROS production. The NF-κB signaling is crucial for regulating cell proliferation, apoptosis, and inflammatory responses [42]. After DHCA, the transcriptional activity of NF-κB was increased [1, 5]. Consistent with our findings, a previous study has demonstrated that TPL attenuated neural apoptosis by inhibiting the NF-κB pathway in cerebral I/R injury rats [13]. Thus, the protective effects of TPL against DHCA are possibly related to its activation of the Nrf2 pathway and inhibition of NF-κB activity.

## CONCLUSIONS

Together, our data demonstrate that TPL improved neurobehavioral functions, inflammation, oxidative stress, and neurotrophin levels in DHCA rats. TPL also attenuated microglia activation and cell death in DHCA rats. TPL-mediated Nrf2 signaling and NF-κB p65 activity might be potential molecular mechanisms underlying the neuroprotective effects of TPL in DHCA rats. As a biologically active compound, TPL expands the horizons of treatment tactics to avert or restrict neuroinflammation and related neurocognitive obstacles after DHCA.

## MATERIALS AND METHODS

### Animals and drug treatments

All animal experiments and operations complied with the ARRIVE guidelines and were performed in accordance with the National Institutes of Health guide for the care and use of laboratory animals [43]. This study was approved by the Research Ethics Committee of Fujian Medical University (Approval No. 2018-056). Sixty 12–14 weeks old male Wistar rats were obtained from Vital River Laboratory Animal Technology Co., Ltd. (Beijing, China). Animals were randomly assigned into 6 groups: (1) control (rats without any intervention, n=10); (2) sham (rats were cannulated without exposure to DHCA, n=10); (3) DHCA (n=10); and (4–6) TPL (DHCA + TPL, three subgroups of different dosages, n=10). TPL was purchased from Sangon Biotech. Co., Ltd. (Shanghai, China) and dissolved in pure dimethyl sulfoxide (DMSO) as a stock solution. The DHCA group and DHCA + TPL groups received intravenous (iv) DMSO or different doses [44] of TPL (100 μg/kg, 200 μg/kg, and 300 μg/kg, respectively, iv) 20 min before DHCA, and then were treated for 7 days in succession when DHCA had finished. Rats were housed at normal ambient room temperature (20 ± 2° C) under a 12 h:12 h light:dark cycle with free access to food and water.

### Cardiac surgery with CPB/DHCA

**Figure 8:**
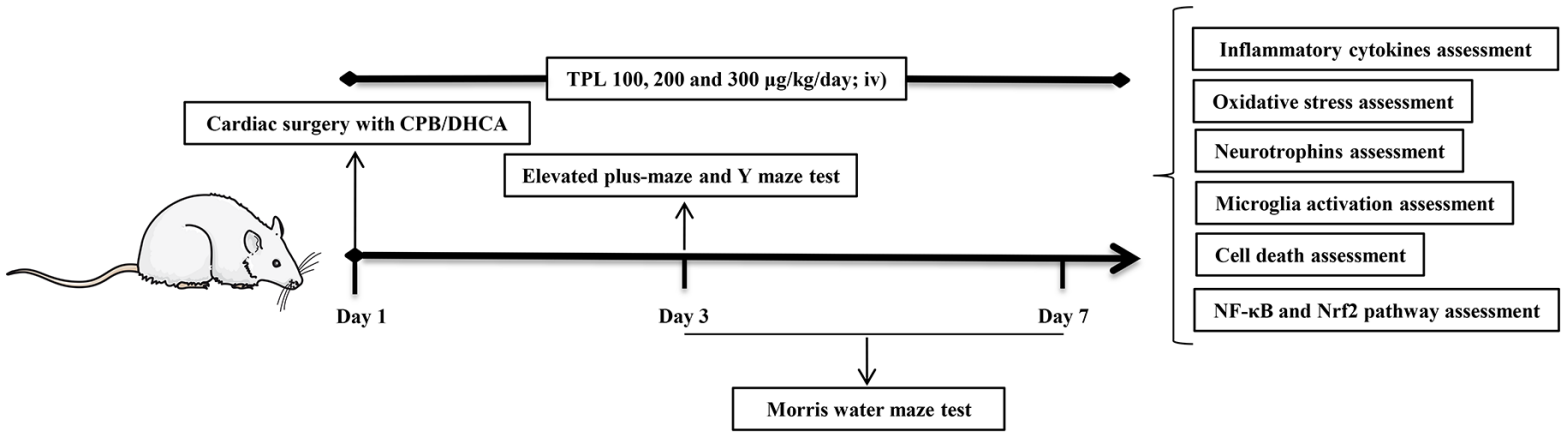
**Schematic diagram of treatment schedule and protocol design.** Rats received 100 μg/kg, 200 μg/kg and 300 μg/kg TPL (iv) 20 min before CPB/DHCA and then daily (iv) up to day 7 post operation. On day 3 post operation, rats underwent elevated plus-maze test and Y maze test. From day 3 through day 7 post operation, rats underwent Morris water maze test. After Morris water maze test on day 7 post operation, rats were euthanized and collected plasma and brain tissues to prepare further assessments.

As described in previous studies [1], after fasting for 12 h, the animals were anesthetized with 2% sevoflurane. Mechanical ventilation was initiated after inserting a 14-G intubation into the trachea. Then 200 IU of heparin and 6 μg of fentanyl was administered via iv using an arterial catheter in the rats’ tail artery. CPB was initiated at a flow velocity of 140–160 ml/kg/min. When rats experienced hypothermia for 30 min to the determined pericranial temperature of 16–18° C, the flow rate was reduced to 50% and CPB was stopped. DHCA was started and confirmed by electrocardiographic asystole, without any measurable mean arterial pressure (MAP). After 1 h of DHCA, CPB was resumed until the pericranial temperature increased to 34° C for 30 min, and then mechanical ventilation was restarted for 2 h. The rats were placed in a warm and oxygen-rich environment and observed for 6 h after the operation. After the final neurobehavioral functions tests were completed on day 7, animals were euthanized. The plasma and brain were collected to prepare for further experiments (Figure 8).

### The EPM test

The EPM test was used to determine anxiety-like behaviors. The protocol of this test was based on a standard protocol reported by previous studies [45]. The EPM consisted of two open (50 cm×10 cm) arms and two closed (50 cm×10 cm×40 cm) arms, which connected to the central platform (10 cm×10 cm). The entire maze was raised to a height of 50 cm above the floor. The animals were placed on the center platform facing an open arm for 5 min. The percentage of open-arm entries and open-arm time were measured.

### Y-maze test

The Y-maze test was used to determine working memory and consisted of three arms (40 cm×4.5 cm×12 cm) placed at 120° to each other. First, the rats were placed in the arm and the times of each rat entering the arms within eight minutes were recorded. Successively entering a new branch before returning to the two previously visited branches was defined as successful alternation. The percentage of correct alternations was calculated according to a previous study [46].

### MWM test

The MWM test was performed from day 3 to day 7 to evaluate spatial learning and memory after DHCA. The water maze consisted of a round pool with a diameter of 1.5 m and a height of 0.3 m, corresponding cameras, and the data analysis software Ethovision XT 11.5 (Noldus, Netherlands). The pool was divided into 4 quadrants on average, and a black platform with a diameter of 0.12 m that was approximately 2 cm beneath the water was randomly located in a quadrant of the pool. Rats were trained to find the submerged platform starting from the four quadrants, every day for a total of 4 days. In each trial, the maximum latency for rats to reach the platform was 90 s. If the rat did not find the platform it was guided there. The rats were allowed 15 s to stay on the platform. Additionally, the rats were tested in a cued trial of the water maze to assess their ability to swim to a platform in the maze. In this, the latency and speed of rats to swim to a platform that was made visible and cued was used to rule out potential influences of treatment on behaviors that were not directly related to cognition. On probe day (day 7), the hidden platform was removed, and the rats were thrown into the water at any selected point of a pool quadrant. The time spent in the target quadrant and the swimming speed were then recorded.

### Measuring inflammatory cytokine levels

Hippocampal tissues were isolated and homogenized in ice-cold homogenization buffer supplemented with protease inhibitor (Roche, Basel, Switzerland). The tissues were extracted at ratio of 100 mg of tissue to 1 ml of buffer. The tissues were sonicated and subsequently centrifuged at 10,000 g for 10 min at 4° C. The supernatants were then collected for analysis. TNF-α, IL-6, and IL-1β levels in plasma and brain homogenates were measured by ELISA kits obtained from Boster (Wuhan, China), following the manufacturer’s instructions. All assays were run in duplicate.

### Detecting oxidative stress

Levels of SOD, ROS, MDA, and GSH in plasma and brain homogenates were detected with commercial test kits from Nanjing Jiancheng Bioengineering Institute (Nanjing, China) according to the manufacturer’s instructions. All assays were run in duplicate.

### Measuring levels of neurotrophins

Brain homogenates were used to detect brain-derived neurotrophic factor (BDNF), nerve growth factor (NGF), neurotrophin-3 (NT-3), and neurotrophin-4 (NT-4) levels using ELISA kits obtained from Boster (Wuhan, China) according to the manufacturer’s instructions. All assays were run in duplicate.

### Immunofluorescence staining of microglia

Brain tissues were fixed in 4% paraformaldehyde, embedded in paraffin, and then sectioned into 10-μm thick brain slices, which were used for microglial activation assay. Briefly, slices were incubated with 15% goat serum (Dingguo Changsheng Biotech Co., Ltd., Beijing, China) for 60 min, and then incubated with rabbit anti-Iba1 antibody (1:300, Wako, Japan) overnight. The slices were then incubated with FITC-conjugated donkey anti-rabbit IgG antibody (Abcam, Cambridge, UK, 1:300) for 1 h. The mean fluorescence intensity (MFI) of Iba1 in the hippocampus and cortex were detected using a N-800F fluorescence microscope (Novel, China). All results were quantified using ImageJ software.

### Cell death assessment

Cell death was determined using a fluorescence-based TUNEL technique. TUNEL staining was performed with the One-step TUNEL apoptosis assay kit (Beyotime, China). Briefly, slices were deparaffinized, rehydrated, and then treated with proteinase K for 20 min at 37° C. After three washes with PBS, the slices were treated with labeling solution including terminal deoxynucleotidyl transferase (TDT), fluorescein-dUTP, and buffer at 37° C for 1 h. After 3 washes with PBS, the numbers of TUNEL-positive cells were counted using a fluorescence microscope.

### Western blot analysis

The concentration of total extracted protein was measured using a BCA kit (Dingguo Bio, China). Western blot analysis was performed using 8%–15% SDS/PAGE gradient gels and transferring onto PVDF membranes (Millipore,MA, USA). Antibodies against Nrf2, NQO-1, HO-1, p- P65, P65, and β-actin (1:1000, all from Cell Signaling Technology, Burlington, MA, USA) were used for detection. Immunoreactive bands were developed using Super ECL Reagent (HaiGene, China).

### Statistical analysis

Data were analyzed with SPSS software v17.0 (SPSS Inc., Chicago, IL, USA). All values are presented as $\bar{x}\pm s$. MWM data were calculated by repeated measurements ANOVA, with time as the repeated measure and Fisher’s least significance difference *post hoc* test. The normal distribution of data was confirmed using the Shapiro–Wilk test. Parametric values were analyzed by one-way ANOVA followed by Bonferroni's multiple comparison tests. The Kruskal–Wallis test followed by Dunn’s multiple comparison was used to analyze nonparametric values. A difference with *P*<0.05 was used to indicate statically significant results.
